# The analgesic efficacy of pregabalin for shoulder Arthroscopy: A meta-analysis of randomized controlled trials: Erratum

**DOI:** 10.1097/MD.0000000000027606

**Published:** 2021-10-29

In the article, “The analgesic efficacy of pregabalin for shoulder Arthroscopy: A meta-analysis of randomized controlled trials”,^[1]^ which published in Issue 100, Volume 38 of *Medicine*, Chunhong Liu and Ke Qiann have been added as authors.
